# Tools for collecting information on irregular migration estimates and indicators

**DOI:** 10.12688/openreseurope.20695.1

**Published:** 2025-07-04

**Authors:** Carlos Vargas-Silva, Arjen Leerkes, Denis Kierans, Lalaine Siruno, Albert Kraler

**Affiliations:** 1 Centre on Migration, Policy and Society, University of Oxford, Oxford, England, OX2 6QS, UK; 2 UNU MERIT, Maastricht University, Maastricht, The Netherlands; 3 Department for Migration and Globalisation Research, University for Continuing Education Krems, Krems, Austria

**Keywords:** irregular migration, illegal migration, smuggling, databases

## Abstract

This paper discusses the tools used to collect quantitative data related to irregular migration stocks and flows of the Measuring Irregular Migration and Related Policies (MIrreM) project. The ultimate goal of this exercise was to construct two databases that provide an inventory and a critical appraisal of estimates and indicators related to irregular migration in the countries covered by MIrreM (12 EU member states, the UK, Canada, the USA and five transit countries). The databases contain estimates on the size and characteristics of the irregular migrant population in a given country and the changes in that population, with one database focussing on irregular migrant stocks and the other on flows. The flows database also contains an inventory of other indicators of irregular migration (e.g. border apprehensions). MirreM is a follow-up project to the Clandestino project which covered the period 2000–2007. MIrreM covers the period 2008 to 2023. MIrreM guidelines were adjusted from those developed by the Clandestino project to maintain some consistency across projects, but also to account for changes across the different periods and overall purposes of the projects. In addition, the approach to assessing the quality of estimates and indicators was refined, notably by explicitly distinguishing between statistical indicators, on the one hand, and estimates, on the other, developing different assessment criteria, and collecting information on the use of these data in policymaking. Beyond the immediate purpose of guiding data collection and analysis within the MIrreM project, these tools may also be useful for other researchers working on comparable topics characterised by a lack of robust research-driven data, hard-to-reach target groups and limited and imperfect administrative data.

## 1. Introduction

This paper documents the methodological tools used for the collection of quantitative data related to irregular migration developed in the framework of the Horizon Europe project “Measuring Irregular Migration and related Policies” (MIrreM). The goal of this exercise was to construct two databases that together provide the most complete inventory and a critical appraisal of estimates and indicators related to irregular migration in the countries covered by MIrreM (12 EU member states, the UK, Canada, the USA and five transit countries). The databases contain estimates on the size and characteristics of the irregular migrant population in a given country and the changes in that population, as well as an inventory of other indicators of irregular migration (e.g. border apprehensions).

MirreM is a follow-up project to the EU-funded Clandestino project which covered the period 2000–2007 ^1– 3^. In MIrreM, we cover the period 2008 to 2023. MIrreM guidelines were adjusted from those developed by the Clandestino project to maintain some consistency across projects, but also to account for changes across the different periods and overall purposes of the projects. In addition, the approach in assessing the quality of estimates and indicators was refined, notably by explicitly distinguishing between statistical indicators, on the one hand, and estimates, on the other and developing different assessment criteria.

The collection of estimates and indicators was the result of desk research by local research teams in each country. In addition to academic outputs, the teams reviewed government reports, think-tank publications and websites of national statistical offices. Moreover, as part of other MIrreM activities researchers interviewed different stakeholders at the local and national level. During these interviews, researchers asked about the existence of estimates and indicators of the irregular migrant population as well as their use in policymaking.

The discussion below is divided in three sections. First, we tackle the issue of defining irregular migrants. Second, we explain the collection and assessment of irregular migration *estimates*. Third, we explain the collection and assessment of irregular migration *indicators*.

## 2. Who counts as an irregular migrant?

The first task in creating a database of irregular migration estimates and indicators is to define the target population, that is, to define who counts as an irregular migrant. Defining an irregular migrant is complex and it is difficult to have a unique description of who fits into this category.

For instance, some recent estimates of irregular migration in Europe have included asylum seekers ^4^. Their importance on estimates is substantial with asylum seekers accounting for a quarter of some estimates of the irregular migrant population of Europe. Including asylum seekers in estimates of irregular migration is controversial. On the one hand, they are still awaiting a decision on their pending application, and are allowed to live in the country while this process is completed ^5^. On the other hand, a majority entered the country without permission, and many are likely to be rejected, leading to possible future deportation. ^1^

While there thus is no doubt that asylum-related migration is somehow interlinked with migrant irregularity, asylum seekers clearly are not unlawfully staying. Indeed, article 31(1) of the Genevan refugee Convention obliges States not to penalise unlawful entry or stay of refugees “provided they present themselves without delay to the authorities and show good cause for their illegal entry or presence.” In a similar vein, rejected asylum seekers and other migrants issued a return decision who have not (yet) returned are unlawfully staying in the sense that they do not possess a legal right to stay and are under an obligation to leave the country of their current residence, but their situation is clearly different from “undetected” migrants in an irregular situation whose presence is not known by migration authorities. In some countries, the obligation to leave may also be formally suspended, if migrants cannot be returned for longer periods of time. While a suspension of removal such as Duldung (“Toleration”) in Germany does not provide a right to stay, it nevertheless provides a legal recognition of the presence of such migrants. In Germany, tolerated migrants may also gain access to work if the situation persists over longer time, thus making toleration very similar to, yet more precarious than temporary residence permits for work ^7^.

In the EU, the situation of EU nationals whose freedom of movements rights are restricted or suspended is somewhat similar. Their situation is clearly different from those of third-country nationals without a right to stay but they are nevertheless subject to some of the same enforcement mechanisms – if they do not comply with residence conditions – and in practical terms, often face similar restrictions in access to rights as third-country nationals in an irregular situation. In conclusion, focusing on undetected migrants in an irregular situation alone would not capture the broader categories of migrants somehow implicated in irregular migration, while at the same time the expansion of the category of irregular migrant risks extending a label that deligitimises migrants’ residence to categories that might in fact have reasonable claims to residence. In MIrreM, we have resolved this tension by introducing ‘related categories’ – rather ‘classes’ within a systematic classification system, in addition to the ‘class’ of migrants in an irregular situation. Together, these categories can be conceptualised as migrants with a precarious legal status ^8^. Migrants with a precarious legal status can be defined as those “individuals who lack regular immigration or residence status or, having a conditional or temporary status, are vulnerable to the loss of that status. They are therefore deprived of or run the risk of losing most basic social rights and access to services” ^9^.

While we follow this conceptual approach, for measurement purposes and considering the variety of definitions used for existing estimates and indicators of irregularity, it is essential to decompose the broad category of “migrants” with a precarious legal and identify specific “classes” within this broad category.

In the following, we discuss the different “classes” we identify. We use the terms “category” and “classes” interchangeably when it comes to overarching categories (irregular migrants, migrants with a provisional status, and EU migrants with restricted freedom of movement rights) to denote that the boundaries between these three groups are not entirely clearcut. Yet our main concept is “classes”, following our ambition to build a systematic classification system. While categorisation (and therefore categories) refers to the “process of dividing the world into groups of entities whose members are in some way similar to each other” without clear inclusion/exclusion criteria ^10^, classification “involves the orderly and systematic assignment of [an] entity to one and only one class within a system of mutually exclusive and non-overlapping classes”.

Building on the Clandestino definition of the irregular migrant population, in MIrreM the target population of irregular migrants are defined as ^11^:

- Those without any legal residence status in the country they are residing in.
- Those, although possessing an authorisation of some sort whose presence in the territory – if detected – may be subject to termination through an order to leave and/or an expulsion order because of their activities.

The latter, for instance, include visa-free citizens engaging in work, students working more than allowed or persons with falsified documents.

We are also interested in data regarding status situations that – in some respects – are comparable to the situation of irregular migrants, defined in an EU context as irregularly staying third country nationals. These “related status situations” are part of what we conceptualise as “precarious statuses” ^9^ include (but are not limited to):

- EU citizens from other EU Member States who are at risk of being issued a removal order and/or residence ban on public order grounds or a criminal charge.
- EU citizens who do not meet the residence requirements of the Citizens Directive (Directive 2004/38/EC), notably the sufficient means requirements and who do not yet enjoy the right to permanent residence.
- Third-country nationals whose removal has been formally suspended (“Duldung”/Toleration in DE).
- Victims of trafficking from third countries holding a temporary permit on grounds of trafficking.
- Unaccompanied migrants who may enjoy protection from expulsion despite an unsuccessful asylum claim.
- Individuals that may in principle be entitled to residence but have not obtained a residence title (e.g. children of legal migrants who don’t possess a resident permit because their parents have failed to obtain one at birth).

We interpret asylum as linked, but not coterminous with irregular migration. We are interested in related flows (e.g. negative decisions, absconding or termination of procedures, which in turn may signal absconding or onward migration). Similarly, we are interested in asylum applications as potential indicators of irregular entry.

Based on this reasoning we focus on three types of situations – (1) migrants in an irregular situation; (2) migrants with a provisional status or a reasonable claim to a provisional status, and (3) EU citizens from other EU Member States without a right to residence as explained in more detail in Table 1. While only the first category strictly concerns migrants in an irregular situation, each of the other two categories are important to consider.

**Table 1.** Definitions of irregular migrants and related categories.

Category	Definition	Examples
Migrants in an irregular situation	Includes: a) third-country nationals (i.e. non-nationals in CA, US, UK) without any legal residence status in the country they are residing in, and b) Persons engaged in an activity that violates the terms of their permission to remain the country and if detected could result in the revocation of their permission to remain in the country and/or their expulsion from it.	Third-country nationals (non-nationals in CA, US, UK) without any status
		Students working more than allowed
		Unregistered persons with false papers and identities
		Persons issued with a return decision who are not removed.
Migrants with a provisional status or a reasonable claim to a provisional status	Third-country nationals (i.e. non-nationals in CA, US, UK) who enjoy a provisional right to stay subject to a review of their case	Persons whose removal has been formally or informally suspended
		Individuals awaiting status determination
		Unaccompanied minors whose asylum claim has been rejected
		Third country (non-national) victims of trafficking with a provisional permit to stay
EU citizens from another EU MS without residence rights	EU nationals who do not or no longer enjoy the right to movement and/or settlement in the EU and are liable to be removed because they do not meet residence conditions or are subject to restrictions of free movement rights.	EU nationals with a residence ban on public order grounds or criminal charges
		EU citizens without long term residence and without sufficient means

Source: Authors’ own analysis.

We include migrants with a provisional status or a reasonable claim to a provisional status – category (2), as per above – because they affect the stock of irregular migrants. That is, rejected asylum seekers add to irregular migrant stock and migrants whose removal is suspended (whether through a mere suspension or a temporary residence permit) on human rights or other grounds reduce it. Furthermore, we include migrants with a reasonable claim to a provisional status to account for migrants who do not (yet) have formal proof of their provisional status. Asylum seekers waiting for their first interview, who have not yet been registered as asylum seekers, and have not yet received relevant documents are counted in this category. Likewise, migrants whose removal has been de facto suspended, but whose suspension is not officially documented, fall into this category.

The other category (3) is EU citizens without a right to residence. While their citizenship status as EU citizens and governance through a distinct body of law on the EU level ^2^ clearly distinguishes them from third-country nationals, they are nonetheless subject to similar rights restrictions and enforcement measures. In addition, they feature prominently in some of the data on enforcement measures at the national level (notably voluntary and forced returns).

We note that available data and estimates may not fit into these three categories perfectly. For example, data on migrants found to be illegally employed could mean they were not entitled to work or that they were in informal employment, where no taxes or social security contributions were paid. The data may not allow differentiation, for example, between legal categories, when data on workplace apprehensions of workers employed in breach of employment, tax, social security, migration or other laws does not distinguish between different statuses. 

In addition to distinguishing different categories of the migrant population with a precarious status, we also distinguish between different pathways into and out of irregularity, building on the demographic balance model proposed by the Clandestino project ^12^. The Clandestino demographic model of migrant irregularity distinguished between stocks of irregular residents (and different subgroups within the stocks of irregular residents without, however aiming to identify mutually exclusive distinct ‘classes’) and different types of ‘flows’ (demographic, geographic and status-related flows) that increase or decrease the stock of the irregular migrant population. 

We argue that such a demographic approach based on the distinction between population stocks and population flows is not only appropriate for measuring the size of the irregular migrant population and other categories of migrants with a precarious legal status, but it also is a helpful for assessing available statistical indicators and estimates to the different components of the model and identifying for example, for which type of flows data traces or estimates exist and where information is missing. As noted, in MIrreM we refined the Clandestino model by distinguishing between different ‘classes’ within the different flows (or pathways into and out of irregularity) and the three main types of stocks (irregularly staying third-country nationals, migrants with a provisional status, and EU migrants without a right to stay). In addition, we also added the dimension ‘visibility’ to assess whether particular ‘classes’ are known to migration authorities, that is, whether they have been registered and thus data might exist.

**Figure 1.**
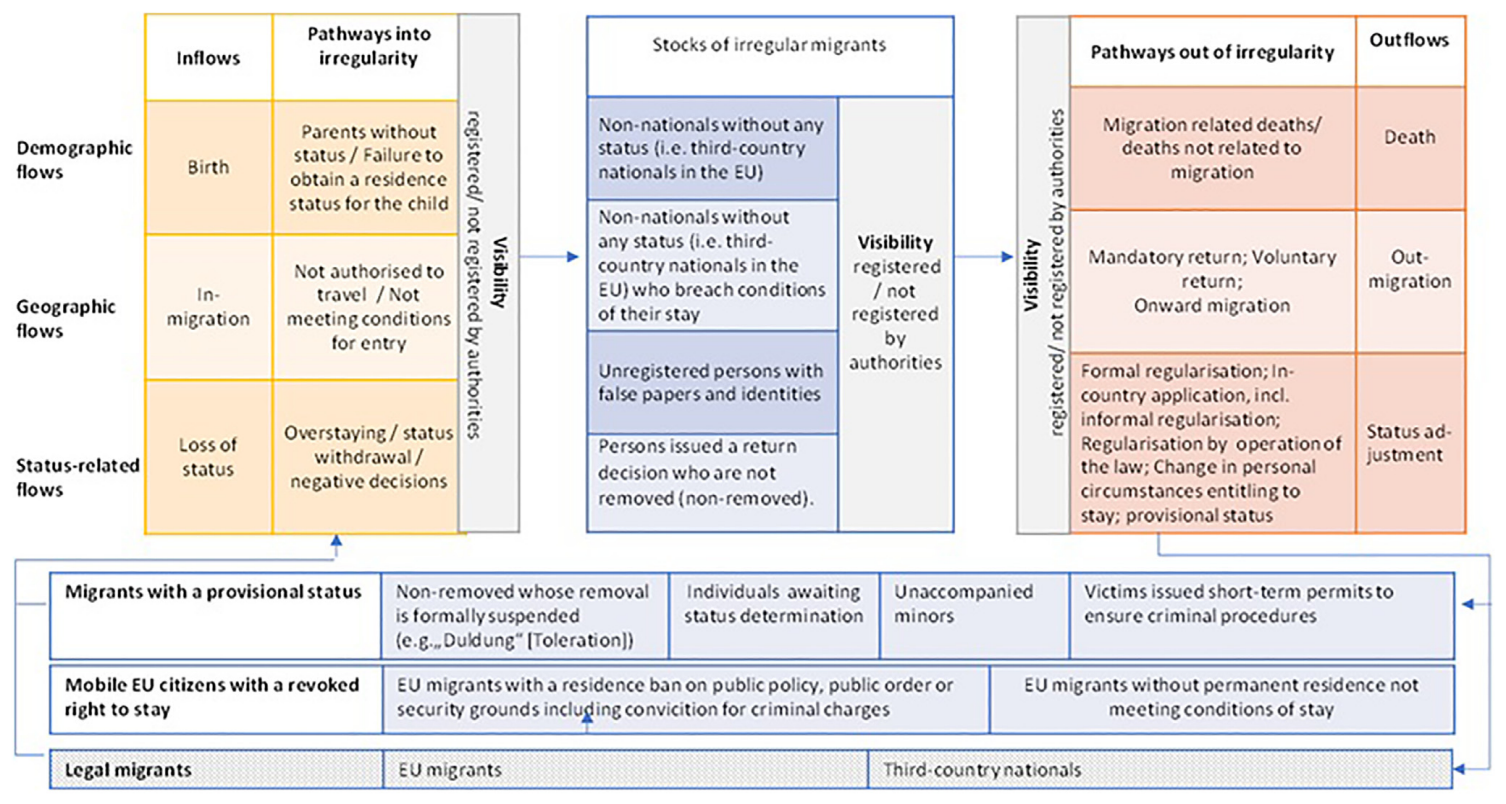
Taxonomy of migrants with a precarious legal status. Source: Kraler and Ahrens (2023).

The resulting ‘taxonomy’ of migrants with a precarious legal status is presented in Figure 1, overleaf.

## 3. What type of quantitative data did we collect?

In MIrreM we are interested in both estimates and statistical data on migrants with a precarious legal status, including migrants with an irregular status. For our mapping of ‘quantifications’ of irregular migration we distinguish between estimates (e.g. the number of irregular migrants that are thought to have been in or entered a country in a particular year) and statistical indicators, which may contribute to an estimate (e.g. border apprehensions). As such, we have developed separate assessment tools that reflect the differences between these two types of quantifications.

We define estimates as any figures that have been produced following some sort of ‘methodological’ procedure. For the purpose of the MIrreM project, 'methodological procedure’ simply means that a figure is based on some form of systematic and reasoned approach, which includes both lay /stakeholder-based estimates (e.g. an estimate of the number of irregular migrants in a city based on client data and some multiplier which a stakeholder considers plausible for one or another reason) as well as estimates based on more sophisticated scientific approaches. Estimates may often be based on statistical indicators, but are not synonymous with these. Finally, we also subsume population estimates based on surveys (that exist in some countries) under estimates.

By contrast, we define statistical indicators as any statistical data derived from “counts of events linked to persons or counts of persons at a certain reference date or period” or counts derived from registers, that is “regularly or continuously updated data systems containing information about a person’s current status” ^13^. In contrast to simple counts based on aggregation of a limited number of indicators, registers can be exploited more flexibly, provide information on trajectories and a person’s status at any time and increasingly can also be linked with other registers. Example of (simple) counts of ‘events’ include statistics on border apprehensions or refusals at a border, statistics on return decisions, etc. An example of counts of persons would be the number of undocumented patients treated by specialised health services. Register-based information will be only available for persons who are in some sort of procedure and are thus registered by authorities, for example persons whose removal is suspended.

## 4. How did we collect and assess data on *estimates* of irregular migration?

MIrreM collected the information on estimates of irregular migration using a series of tables to be filled in by local research teams in each country.

Table 2 shows the reporting matrix for irregular migration estimates. Teams completed a different table for each estimate, including sub-categories. For instance, if the stock of irregular migrants was also divided across genders, then teams completed one table for the total irregular migrant population and one for each gender category. Note that the goal of MIrreM was to have the most complete dataset regarding irregularity in participating countries. Therefore, teams reported all sub-categories that were available.

**Table 2.** Reporting matrix for each estimate of irregularity.

Type	Period (and frequency)	Central estimate	Range	Group	Datasets used
**Quality assessment rubric**					
	1 to 3 points each		Explanation		
Accessibility					
Documentation					
Replicability					
Methodology					
Data					
**Background information:**					
**Link:**					
**Use in policymaking:**					

Source: Authors’ own analysis.

The information collected is contained in 14 and 15.

The discussion below includes the information contained in each space of the table.

### 4.1 Type

This space is for indicating whether it is a stock or flow estimate being recorded. Stocks refer to the total population of irregular migrants at a specific point in time (e.g. 1 January 2023). As explained below ( 4.5 and Table 3), this estimate could be for the whole country or specific cities/regions. Flows refer to the changes in the stock of irregular migrants, for instance, the number of irregular migrants entering (inflows), or leaving (outflows) an area (e.g. country, region, city) in a given year or other period (see 3.2). The two databases that emerged are distinguished as follows: one database focuses on stocks ^14^, while the other examines flows ^15^.

**Table 3.** Non-exhaustive list of groups (i.e. composition).

Groups
All
By gender
By nationality
By country of birth
By previous country of residence
By category of entry (e.g. tourist visa, student visa. Irregular entry, family visa, by birth)
By location (e.g. city, region, port of entry)
By age group
By economic activity (e.g. employed, unemployed)
By economic sector (e.g. construction)
Any other sub-categories

Source: Authors’ own analysis.

### 4.2 Period (and frequency)

Here, we asked national teams to report on the year or period related to the estimates and the frequency with which these estimates are published. Remember that the goal of MIrreM is to cover the period from 2008 to 2023. Any estimates that only cover a period before 2008 were not included. However, any estimates that cover the post-2008 period were included even if also included information from before 2008. For instance, if there is an estimate that covers the period 2006 – 2016, then those were included in the reporting. The data was reported in the smallest unit of time available (i.e. highest frequency). In particular, if there is annual data, then the estimates were reported for each year.

### 4.3 Central estimate

Estimates of irregularity often provide a central estimate and a range of values (minimum and maximum, see 4.4). Teams reported the central estimate in this space.

### 4.4 Range

Estimates of irregularity often provide a central estimate (see 4.3) and a range of values, sometimes called the margin of error. We asked teams to report the range of values in this space. The lower value of a range estimate indicates it is likely that there are at least this many irregular migrants (also referred to as a conservative estimate). The upper value of a range estimate indicates it is likely that there are at most this many irregular migrants (i.e. maximum).

### 4.5 Groups

This field refers to the group of the population that is linked to the estimate. In some cases, estimates can refer to the total number of irregular migrants (i.e. all), but in other cases it refers to particular groups. In MIrreM, we were interested in collecting information on all possible groups for which there is information on status precarity (i.e. our three overarching categories described above under Section 2), but had particular interest in disaggregation by gender, nationality and age.

If the estimates include a total estimate (e.g. number of irregular migrants in the UK) and estimates for a particular group (e.g. number of irregular migrants in London), teams started with a table for the broader category and then included tables for the groups. The idea was to always go from the broader estimate to the narrower estimate. For example, you can go: ( Table 1) number of irregular migrants in the UK, ( Table 2) number of irregular migrants in London, ( Table 3) number of irregular children in London.

Table 3 provides a non-exhaustive list of possible groups for which estimates could be available. Please note that some estimates might combine two groups (e.g. gender and nationality).

### 4.6 Datasets used

In this space we collected information on the datasets that were used for the estimates. Table 4 includes a non-exhaustive list of possible datasets. If multiple datasets were used, we asked teams to include all of them.

**Table 4.** Possible datasets and sub-categories.

Datasets
Enforcement data (data from border guards, police, labour market inspection units, etc.)
Regularisation data (amnesties, continuous regularisation programmes, etc.)
Service access data (health services, schools registers, etc.)
Other administrative data
Census/ general survey
Expert survey
Migrant survey
Employer survey
Unknown

Source: Authors’ own analysis.

### 4.7 Quality

The quality assessment for the estimates of irregular migration was influenced by the previous approach of the Clandestino project and more recent related methodological discussions ^16– 18^, as well as the FAIR principles for data management.

In Clandestino the quality assessment consisted of four categories (high, medium, low, and low with plausibility warning). Each of those quality assessments combined multiple aspects such as quality of data, documentation and estimation technique. For example, an assessment of high for absolute numbers was a described as: “ *Estimate fulfilling usual academic standards: full documentation, comprehensive and consistent, limitations clearly indicated*” ^19^.

MIrreM used a more disaggregated approach in order to allow for more nuance in the quality assessment. The valuation differentiates quality across five categories: accessibility, documentation reliability, methodology, and data. In each of those categories it was possible to assign a score from 1 (low) to 3 (high). Table 5 includes the explanation of the scoring for each of these categories.

**Table 5.** Criteria for quality evaluation of estimates.

Criteria	High (3 points)	Medium (2 points)	Low (1 point)
Accessibility	All raw data used to construct the estimate is publicly available and electronically accessible with no permissions required.	At least some of the raw data used to construct the estimate is only available on request from relevant authorities. If some of the data is not available at all, then give 1 point.	At least some of the raw data used to construct the estimate is not available for most potential users.
Documentation	Full documentation about data and methods are available and accessible. The level of information allows for replication of the estimates.	Limited information on data, estimation methods, and quality are available and accessible. Insufficient details to replicate the estimates.	Information on data and estimation methods is neither available nor accessible.
Reliability	Analysis includes demonstrated reliability indicators, with limitations clearly specified (e.g. ranges, alternative calculations, characterisation as minimum or maximum estimate).	Some discussion of reliability, but no indicators in quantitative terms.	Missing a discussion of reliability.
Methodology	Methodology is adequate and comprehensive including, but not limited to, rigorously implemented multiplier or residual studies.	Methodology is adequate, even if not comprehensive, including but not limited to: (1) Simple multiplier calculations; (2) Simple residual estimates; (3) Adjustment of older estimates with partly insufficient data; (4) Aggregate estimates for different groups, partly relying on plausibility calculations.	Inadequate method and application of the method; resulting estimate lacks foundation
Data	The analysis relies on an adequate dataset not likely to have a considerable bias, including no bias for any group estimates. There are no strong assumptions regarding the data.	The analysis relies on a biased dataset. There are plausible adjustments and assumptions. This includes cases in which the dataset does not provide the information necessary or it is necessary to make strong assumptions.	The analysis relies on a biased dataset, without proper adjustments. The assumptions regarding data are not plausible.

Source: Authors’ own analysis.

### 4.8 Background information

This space is for indicating the type of person or institution supplying the estimate and explains the estimation procedure. The estimation procedure was explained in four of five sentences only. In particular, we were interested on the main methodological approach (e.g. residual method) and any details that are relevant or key assumptions contained in the analysis.

If there are two estimates that come from the analysis (e.g. total estimate and group analysis), teams explained the methodology in the first table and referred to that table in sub-sequent tables.

In addition, we asked teams to provide a full reference to the study in this section.

### 4.9 Link

A link to the main document related to the estimate (e.g. report or academic paper) should be noted here. If the document was not available online, teams were asked to make a note of this.

### 4.10 Use in policymaking

We asked teams to reflect on how the estimate is used in policymaking. This can range from not used at all to being used as a key measure of success of policies related to irregular migration. It was also possible to indicate the extent to which officials and others view the estimate as trustworthy and to include examples of policy or programming where the estimate was (or was not) cited or otherwise used.

### 4.11 Examples

Table 6 and Table 7 include some examples of possible reporting for different datasets using UK information.

**Table 6.** Example 1 estimates.

Type	Period (and frequency)	Central estimate	Range	Group	Datasets
Stock	April 2017 (ad hoc, unrepeated)	674,000	Low = 594,000, High = 745,000	Total irregular migrant population in the UK	1. Census 2. Annual Population Survey 3. International Passenger Survey 4. Mortality data 5. Visa data
**Quality assessment rubric**					
	1 to 3 points each		Explanation		
Accessibility	3		The study relies on publicly available data.		
Documentation	3		The study includes documentation explaining each step of the analysis.		
Reliability	3		The study includes a range for the estimate (i.e. low estimate, high estimate) in addition to the central estimate.		
Methodology	3		The study uses a comprehensive application of a residual method.		
Data	2		The study relies on several datasets that do not have the exact information that is required. For instance, there is no information on how many of those who emigrated had settlement in the UK and is necessary to make assumptions based on related data.		
**Background information:** Authors are academics affiliated to the University of Wolverhampton. The research was commissioned by the Greater London Authority. Full reference: Jolly, A., Thomas, S. & Stanyer, J. (2020). London’s children and young people who are not British citizens: A profile. London, UK: Greater London Authority. The analysis uses the residual methodology. The different steps of the process are explained clearly as well as the assumptions involved in each step. Value ranges are calculated differently for the various sources of data, and when combined comprise the high, low and central estimates. The estimate excludes the UK-born children of irregular migrants. If these are included, the central estimate is 809,000.					
**Link: ** https://www.london.gov.uk/sites/default/files/final_londons_children_and_young_people_who_are_not_british_citizens.pdf					
**Use in policymaking:** The estimates were cited by a range of stakeholders in the UK when giving evidence to parliament (e.g. https://committees.parliament.uk/writtenevidence/39726/html/).					

Source: Authors’ own analysis.

**Table 7.** Example 2 estimates.

Type	Period (and frequency)	Central estimate	Range	Group	Datasets
Stock	April 2017 (ad hoc, unrepeated)	397,000	Low = 350,000, High = 478,000	Irregular migrant population in London	Enforcement data: population of individuals who have been notified of their liability for detention and removal from the UK
**Quality assessment rubric**					
	1 to 3 points each		Explanation		
Accessibility	3		The study relies on publicly available data.		
Documentation	3		The study includes documentation explaining each step of the analysis.		
Reliability	3		The study includes a range for the estimate (i.e. low estimate, high estimate) in addition to the central estimate.		
Methodology	2		The analysis in this table takes the number presented in Example 1 and combines with the proportion of individuals who have been notified of their liability for detention and removal from the UK that reside in London. The analysis assumes that this proposition is a good indicator of the share of the irregular migrant population in the UK that resides in London. This assumption could be correct, but there is no sufficient information to validate it		
Data	2		The study relies on several datasets that do not have the exact information that is required. For instance, there is no information on how many of those who emigrated had settlement in the UK and is necessary to make assumptions based on related data.		
**Background information:** This estimate comes from the analysis presented in Example 1.					
**Link:** https://www.london.gov.uk/sites/default/files/final_londons_children_and_young_people_who_are_not_british_citizens.pdf					
The report that produced these estimates was commissioned by the Mayor of London as part of its Citizenship and Integration Initiative. It has been cited by the Mayor in his lobbying to central government around the EUSS and support programmes to help EU citizens and their families apply for status (see https://www.london.gov.uk/press-releases/mayoral/calls-for-urgent-action-to-support-young-londoners).					

Source: Authors’ own analysis.

## 5. How did we collect and assess data on *indicators* of irregular migration?

Next, we describe the reporting related to the *indicators* of irregular migration. Indicators refers to series such as border apprehensions, expulsion orders, etc. which are not the same as a stock or flow estimate but provide information on irregularity or precarity of legal status, and could be used to develop an irregular migration estimate. Table 8 is the tool to report these indicators, and the information collected is contained in the MIrreM Public Database on Irregular Migration Flow Estimates and Indicators ^15^.

**Table 8.** Reporting matrix for each indicator of irregularity.

Indicators	Period (and frequency)	Count/ average
**Quality assessment rubric**		
	1 to 3 points each	Explanation
Accessibility		
Documentation		
Validity and reliability		
**Background information:**		
**Link:**		
**Use in policymaking:**		

Source: Authors’ own analysis.

The discussion below includes the information that we asked the teams to include in each space of the table.

### 5.1 Indicators

This section is for the indicator that is being reported on. MIrreM is interested in *all* possible indicators of irregularity that are available for each country, including those that are not necessarily transmitted to or collected by Eurostat. However, as a minimum we wanted to have information on the indicators presented in Table 9.

**Table 9.** Required indicators of irregularity (minimum).

Indicator	Explanation
Border apprehensions	Non-nationals (third-country nationals) apprehended or intercepted by authorities while or after attempting to illegally cross a border. In practice, border and inland apprehensions may not be distinguished.
Rejection at the border (Refusal of entry)	Those formally refused entry at the external borders because of failure to fulfil all the entry conditions as laid out in the Schengen Borders Code.
Inland apprehensions	Those found to be illegally present in the territory of the Member State and intercepted by authorities. In practice, border and inland apprehensions may not be distinguished.
Dublin regulation (incoming)	Incoming take charge requests and decisions for reasons of irregular entry or stay; or incoming take back requests regardless of implementation indicating secondary movements.
Births in irregularity	Number of births of babies born without a status. Usually, this concerns children of irregularly staying parents without access to a residence title. It may also concern children of legally resident non-national parents failing to register their child and obtain a residence title.
Visa overstaying	This category encompasses both overstaying of visas is in the narrow sense (Schengen Visa, national visa D) as well as overstaying of residence permits of a longer duration
Withdrawal of status	Rejection of an asylum application; withdrawal of a temporary residence status tied to a particular activity, notably employment withdrawal of a temporary or permanent status after a serious criminal offence or on grounds of public order;
Expulsion orders	Those issued orders to leave the country.
Returns	Those returned from an EU country following an order to leave.
Dublin regulation (outgoing)	Outgoing take back requests and decisions.
Deaths in irregularity	Persons without a residence status who died while without status. Also includes deaths in custody (upon forced removal, in detention pending deportation).
Regularisation	Persons who are individually regularised in cases of hardship or as asylum seekers; persons profiting from a collective regularisation programme.

Source: Authors’ own analysis.

### 5.2 Period (and frequency)

Here, country teams reported the year or period related to the indicators and the frequency with which these indicators are published. Remember that the goal of MIrreM is to cover the period from 2008 to 2023. Therefore, any indicators that only cover a period before 2008 were not included. However, any indicators that cover the post-2008 period were included even if also include information from before 2008. Teams reported the data at the highest frequency for which it is available.

### 5.3 Count/Average

In this space we asked teams to include the average of the indicator for the period in question.

### 5.4 Quality

Table 10 includes the information to consider when evaluating the quality of the different indicators. As with the assessment presented in Table 5, this assessment was influenced by the previous approach of the Clandestino project and recent related methodological discussions. In this case there are three criteria: accessibility, documentation, and validity/reliability.

**Table 10.** Criteria for quality evaluation of indicators.

Criteria	High (3 points)	Medium (2 points)	Low (1 point)
Accessibility	Data is publicly available and electronically accessible with no permissions required	Data is available on request from relevant authorities	Data is available, but access and use are exclusive to authorities
Documentation	Sufficient and transparent information on data and methods are available and accessible; a comprehensive quality report is also available	Limited information on data, methods, and quality are available and accessible	Information on data, methods, and quality are neither available nor accessible
Validity and reliability	Data is representative of the phenomenon it is supposed to measure and adequately reflects the type of irregular migration being measured; data is relatively complete (not highly selective) and does not indicate internal contradictions	Data is selective and points to some internal contradictions	Data is neither valid nor reliable

Source: Authors’ own analysis.

### 5.5. Background information

This field asks for a short explanation of the irregular migration flow being measured, the type of person or institution supplying the information on the indicator, and the methodology used. When applicable, teams also provided a full reference to the study in this section.

### 5.6 Link

This space is for supplying a link to the indicator (if available).

### 5.7 Use in policymaking

Teams were asked to describe how the indicator is used in policymaking, ranging from not used at all to its use as a key measure of success of policies related to irregular migration.

### 5.8 Examples

Table 11 and Table 12 include some examples of possible reporting for different indicators using information from the Netherlands.

**Table 11.** Example 1 indicators.

Indicators	Period (and frequency)	Count/ average
Returns (TCNs returned following an order to leave)	2015 – 2021	2015 – 8,630 2016 – 12,430 2017 – 8,390 2018 – 8,980 2019 – 11,185 2020 – 8,870 2021 – 3,200 (preliminary)
**Quality assessment rubric**		
	1 to 3 points each	Explanation
Accessibility	3	Publicly and electronically available data from both by Statistics Netherlands (CBS) and Eurostat, but the latter provides more extensive raw data.
Documentation	3	Adequate documentation with metadata available
Validity and reliability	2	The indicator is a valid measure of geographic outflows, and each person is counted only once within the reference period. However, as indicated in the guidance notes from Eurostat, the figures do not include other concluded return wherein one can reasonably presume that the TCN was returned based on some assumptions. In the case of NL, it is also known that there are cases in which people are included in these return figures despite not having received a return decision, so there is an issue with regard to the way enforced return is registered (Carrera, 2016; Maliepaard *et al.*, 2022).
**Background information:** The figures are compiled by CBS and transmitted to Eurostat quarterly and refer to TCNs against whom a return decision has been issued and where a demonstrable departure to a country outside the EU/EFTA has taken place. Data do not include persons who are transferred to another MS under the Dublin Regulation. The data above are annual sums and can be disaggregated into three types: (1) assisted voluntary return, (2) assisted forced return/enforced return, and (3) non-assisted voluntary return. Data is only available for 2015 to 2021.		
**Links:** https://opendata.cbs.nl/statline/portal.html?_la=nl&_catalog=CBS&tableId=85334NED&_theme=394 https://ec.europa.eu/eurostat/databrowser/view/migr_eirtn1/default/table?lang=en		
**Use in policymaking:** The number of returns is cited in the *State of Migration*, an annual report co-produced by the Dutch Ministries of Justice and Security, Social Affairs and Employment and Foreign Affairs and sent to the Parliament to serve as basis for further development of migration policy. A comprehensive study on return data however, indicates several issues, including political influences as informants have indicated that there have been instances when right wing ministers issued instructions to include certain returns not necessarily covered by the definition to make the figures seem higher (Maliepaard *et al.*, 2022),		

Source: Authors’ own analysis.

**Table 12.** Example 2 indicators.

Indicators	Period (and frequency)	Count/ average
Border apprehensions (TCNs denied entry at the external air and maritime borders)	2019 2020 2021	3,870 (2,900) 2,040 (1,980) 3,280 (3,745)
**Quality assessment rubric**		
	1 to 3 points each	Explanation
Accessibility	3	Data is publicly and easily available
Documentation	2	Data is adequately documented with information on sources and supporting details provided. The figures, however, do not match with those available on Eurostat for the same period, as indicated in the parentheses above). The Eurostat data matches with data from CBS.
Validity and reliability	2	The indicator captures irregular entry and is valid measure of geographic inflows. When cross-checking other sources however, the figures do not match those produced by CBS and then transmitted to Eurostat that are indicated in the parentheses above. CBS/Eurostat also have more extensive data covering 2008 to 2022.
**Background information:** The figures refer to TCNs refused entry at the Dutch borders. They are released by the Dutch government in its annual *State of Migration* report based on data from the seaport police and The Royal Netherlands Marechaussee (military police in charge of safeguarding the security of the State).		
**Links:** https://open.overheid.nl/repository/ronl-2cf0251dee3fec7c64207480c2720226feb4510f/1/pdf/De%20Staat%20van%20Migratie%202022%20-%20DEF.pdf https://opendata.cbs.nl/statline/portal.html?_la=nl&_catalog=CBS&tableId=82268NED&_theme=394		
**Use in policymaking:** The number of border apprehensions is cited in the *State of Migration*, an annual report co-produced by the Dutch Ministries of Justice and Security, Social Affairs and Employment and Foreign Affairs and sent to the Parliament to serve as basis for further development of migration policy.		

Source: Authors’ own analysis.

## 6. Conclusion

Despite the considerable increase of interest in academic and policy circles on irregular migration the quantitative estimates and indicators of this phenomenon are dispersed and there is little consistent assessment of their quality. MIrreM brings together these estimates and indicators and provides a unique and coherent mode of assessment. This paper has explained the methodological tools used to bring those estimates together and assess their quality. Our hope is that the tools, methodological approaches, and datasets developed under MIrreM will be valuable to others interested in irregular migration, particularly those focused on collecting and assessing data on irregular migrants and related topics. Beyond this paper, interested researchers are encouraged to consult the databases themselves ^14, 15^ and the Working Papers that analyse their contents ^20, 21^.

## Ethics and consent

This research was approved by the Ethics Committee at University for Continuing Education Krems. Reference number or ethical approval number: EK GZ 45/2021-2024. No human participants were involved in the study.

## Data Availability

The MIrreM Public Database on Irregular Migration Stock Estimates can be found here: https://zenodo.org/records/13856861. The DOI is: 10.5281/zenodo.13856861 ^14^. This includes the data discussed in the article and a README File, which can be accessed alongside the Database, and contains contextual and technical information about the Database. The data is available on license Creative Commons Attribution Share Alike 4.0 International (CC BY-SA 4.0), which permits almost any use subject to providing credit and license notice.
