# Supplementary material for: Tools for collecting information on irregular migration estimates and indicators
Source: Open Res Eur. 2025 Jul 4;5:176. [Version 1] doi: 10.12688/openreseurope.20695.1 (PMC12395125; doi:10.12688/openreseurope.20695.1)
Supplement: Peer review history

# Reviewer report: Reviewer response for version 1

**DOI:** 10.21956/openreseurope.22385.r57347

**Published:** 2025-09-15

**Authors:** Giacomo Solano

**Affiliations:** 1 Radboud University, Nijmegen, The Netherlands

This paper provides a clear, methodologically grounded framework for collecting, assessing, and classifying quantitative data on irregular migration within the Horizon Europe *MIrreM* project. It succeeds in documenting the tools, definitions, and procedures used to build comprehensive databases of estimates and indicators of irregular migration across a wide set of countries. The contribution is highly relevant for migration studies, policymaking, and comparative research.

Are sufficient details provided to allow replication of the method development and its use by others?

Yes

Is the description of the method technically sound?

Yes

Are the conclusions about the method and its performance adequately supported by the findings presented in the article?

Yes

Is the rationale for developing the new method (or application) clearly explained

Yes

If any results are presented, are all the source data underlying the results available to ensure full reproducibility?

Yes

Reviewer Expertise:

Quantitative analysis of migration; data on migration; indicators.

I confirm that I have read this submission and believe that I have an appropriate level of expertise to confirm that it is of an acceptable scientific standard.

# Reviewer report: Reviewer response for version 1

**DOI:** 10.21956/openreseurope.22385.r56642

**Published:** 2025-09-08

**Authors:** Natascha Zaun

**Affiliations:** 1 Leuphana University of Lüneburg, Lüneburg, Germany

The article describes the Measuring Irregular Migration and Related Policies (MIrreM) project. The aim of this project was to construct two data bases that provide an inventory and a critical assessment of the quality of existing datasets in several EU member states and associated countries. The article presents the approach used for constructing the dataset and describes what it covers and what not. The data base can be seen as the successor of the Clandestino Project which had a similar aim, but only covered the years 2000-2007. MirreM covers the period 2008-2023 and thus takes the issue up where the Clandestino project stopped. 

 Data on irregular notoriously hard to assess for researchers, as irregular migrants are in many cases not registered and scholars have often argued that this leads numbers on irregular immigration often to be used for political purposes (e.g., Vollmer 2011). Having an updated version of the Clandestino project is therefore extremely important for the scientific community and policymakers alive, especially in times where political discourse on immigration is so politically disruptive and toxic and where there are many misconceptions about the issue. The data base is an extremely valuable tool to allow to assess to what extent data is reliable, which is crucial in the context of false claims about how migration numbers.

 The article mainly presents the data base and it therefore does not have a research question or does not present any data- which is why I focus on evaluating how the data base is presented, how well designed the data base is and how well the article is written.

 The article is very well-written und makes the advantages of the dataset very clear. It makes a clear contribution (see my assessment above) which it embedds very clearly into the literature. It is a particularly strong point of the data set that it does also assess data on related forms of immigration, which in my understanding, was not covered in the Clandestino data set. It's assessments of what the include and why is very nuanced and compelling. 

 Also the taxonomy of migrants with a precarious legal status in figure 1 makes an important contribution, as it demonstrates the multitude of pathways through which immigrants can become 'irregular'. 

 The criteria for categorising data in the project are well-defined and transparent and the methodological approach more generally is very clear and easy to follow.  The examples used to present the data base are also clear and help the reader understand the data base better.   

 Overall, the article presents an important endeavour which will be extremely useful and important for researchers and practitioners alike. The data base is constructed in a way that is methodologically sound and well-explained. So, overall, an important and easily accessible text.

Are sufficient details provided to allow replication of the method development and its use by others?

Yes

Is the description of the method technically sound?

Yes

Are the conclusions about the method and its performance adequately supported by the findings presented in the article?

Yes

Is the rationale for developing the new method (or application) clearly explained

Yes

If any results are presented, are all the source data underlying the results available to ensure full reproducibility?

No source data required

Reviewer Expertise:

migration, EU

I confirm that I have read this submission and believe that I have an appropriate level of expertise to confirm that it is of an acceptable scientific standard.

## References

[1] : Policy Discourses on Irregular Migration in the EU - ‘Number Games’ and ‘Political Games’. *European Journal of Migration and Law* .2011;13(3) : 10.1163/157181611X587874 317-339 10.1163/157181611X587874

# Reviewer report: Reviewer response for version 1

**DOI:** 10.21956/openreseurope.22385.r57344

**Published:** 2025-08-28

**Authors:** Mochamad Kevin Romadhona

**Affiliations:** 1 Universitas Airlangga, Airlangga, Indonesia

The research offers a clear and structured overview of the MIrreM project's methodology and objectives in collecting and evaluating quantitative data on irregular migration. The writing is largely informative and well-organized, providing historical context and distinguishing the project’s contributions in comparison to its predecessor, Clandestino. However, several areas would benefit from further clarification, tightening of language, and improved academic tone.

 Furthermorem this research offers a comprehensive and conceptually robust discussion of the complexities involved in defining and measuring irregular migration and associated precarious legal statuses. It provides a clear rationale for the classification system used in the MIrreM project and demonstrates a deep engagement with the challenges posed by the heterogeneity of migrant statuses, particularly in the European context. The authors successfully integrate legal, policy, and data perspectives and build upon existing models to propose a refined framework that is analytically and practically useful.

 Minor comment

 Clarify whether “migrants whose removal is suspended” are counted as part of the irregular stock in all national datasets or only in MIrreM’s schema.

Are sufficient details provided to allow replication of the method development and its use by others?

Yes

Is the description of the method technically sound?

Yes

Are the conclusions about the method and its performance adequately supported by the findings presented in the article?

Yes

Is the rationale for developing the new method (or application) clearly explained

Yes

If any results are presented, are all the source data underlying the results available to ensure full reproducibility?

Yes

Reviewer Expertise:

my research expertise mainly focusing on the case of International migration issue, Sociology of Health and Illness, and Human Rights,

I confirm that I have read this submission and believe that I have an appropriate level of expertise to confirm that it is of an acceptable scientific standard.

## References

[1] : Living beyond borders: the international legal framework to protecting rights to health of Indonesian illegal migrant workers in Malaysia. *International Journal of Migration, Health and Social Care* .2024;20(2) : 10.1108/IJMHSC-04-2023-0038 227-245 10.1108/IJMHSC-04-2023-0038

# Reviewer report: Reviewer response for version 1

**DOI:** 10.21956/openreseurope.22385.r57340

**Published:** 2025-08-27

**Authors:** Thomas Huddleston

**Affiliations:** 1 University of Liège, Liège, Walloon Region, Belgium

The report provides a clear overview of the construction of irregular migration indicators with breakdowns of the relevant steps and categories. This provides an extremely useful overview for understanding the irregular migration flow estimates and indicators database of the MIrreM project, which has demonstrated its significant innovation in improving the measurement of irregular migration stocks and flows. This project is proving to be an academic and administrative benchmark for statistical data collection.

Are sufficient details provided to allow replication of the method development and its use by others?

Yes

Is the description of the method technically sound?

Yes

Are the conclusions about the method and its performance adequately supported by the findings presented in the article?

Yes

Is the rationale for developing the new method (or application) clearly explained

Yes

If any results are presented, are all the source data underlying the results available to ensure full reproducibility?

Yes

Reviewer Expertise:

Migration and integration data collection and indicator construction

I confirm that I have read this submission and believe that I have an appropriate level of expertise to confirm that it is of an acceptable scientific standard.
